# Epicardial fat tissue in patients with diabetes mellitus: a systematic review and meta-analysis

**DOI:** 10.1186/s12933-019-0807-3

**Published:** 2019-01-10

**Authors:** Yingrui Li, Bin Liu, Yu Li, Xiaodong Jing, Songbai Deng, Yulin Yan, Qiang She

**Affiliations:** Department of Cardiology, The Second Affiliated Hospital of Chongqing Medical University, Chongqing, 400016 China

**Keywords:** Epicardial fat tissue, Diabetes mellitus, Meta-analysis

## Abstract

**Background:**

Epicardial fat tissue (EFT) is the visceral fat distributed along the coronary arteries between the pericardium and the myocardium. Increases in EFT are closely related to the occurrence of diabetes mellitus (DM) and cardiovascular disease. To further understand the link between EFT and DM, we conducted a meta-analysis of the relevant literature.

**Methods:**

We systematically searched electronic databases for studies on EFT performed in DM patients and published up to 30 September 2018. We included data on EFT in a DM patient group and a non-DM control group. We then assessed the effect of DM on EFT by meta-analysis and trial sequential analysis (TSA). All statistical analyses were performed using Stata 12.0 and TSA software.

**Results:**

A total of 13 studies (n = 1102 patients) were included in the final analysis. Compared with the control group, DM patients had significantly higher EFT (SMD: 1.23; 95% CI 0.98, 1.48; P = 0.000; TSA-adjusted 95% CI 0.91, 2.13; *P *< 0.0001). The TSA indicated that the available samples were sufficient and confirmed that firm evidence was reached. According to the regression analysis and subgroup analyses, DM typing, EFT ultrasound measurements, total cholesterol (TC) and triglyceride (TG) levels were confounding factors that significantly affected our results.

**Conclusions:**

Our meta-analysis suggests that the amount of EFT is significantly higher in DM patients than in non-DM patients.

## Background

Diabetes mellitus (DM) is one of the most common metabolic diseases worldwide and is characterized as a metabolic disorder of carbohydrates, proteins and lipids [1]. In recent years, the incidence of DM has gradually increased, becoming a serious public health threat [2, 3]. DM can be divided into type 1 diabetes mellitus (T1DM), type 2 diabetes mellitus (T2DM), gestational diabetes mellitus (GDM) and other types of diabetes. Abnormally accumulated visceral fat is a risk factor for insulin resistance, which can reduce insulin sensitivity, increase the expression and secretion of proinflammatory cytokines in adipose tissue and promote the development of DM and cardiovascular diseases [4–7].

Epicardial fat tissue (EFT) is a visceral adipose tissue that surrounds the myocardium and pericardium. It is one of the visceral fat stores in the body. Previous studies have suggested that measurements of EFT are a substitute for visceral fat [8, 9] EFT can secrete inflammatory factors, such as TNF-alpha, IL-6, adipocytokines, and leptin, via paracrine or endocrine activities [9, 10] to locally regulate the myocardium and coronary artery function and regulate lipid and energy homeostasis in vivo. EFT has the ability to release and uptake free fatty acids and to affect low glucose utilization, which plays an important role in metabolic syndrome and coronary artery disease [11–16]. EFT can be measured by echocardiography, cardiac magnetic resonance imaging, and computed tomography (CT) [17]. Recent studies have confirmed that EFT is associated with obesity, fasting blood glucose levels, insulin resistance, and adiponectin in patients with T2DM, and an increase in EFT was observed in patients with T1DM and T2DM [18–22].

In the past few years, several studies have reported that EFT is abnormally increased in DM patients. However, small sample sizes and potential confounders (such as differences in EFT measurements and DM typing) can affect the strength of previous evidence. Therefore, in this study, we conducted a meta-analysis to evaluate the effect of DM on EFT volume to provide a comprehensive overview of this issue.

## Methods

We conducted this meta-analysis in strict accordance with the PRISMA reporting specifications.

### Search strategy

According to the PRISMA guidelines [23], we developed a detailed search strategy. We systematically searched online databases (PubMed, EMBASE, Cochrane Library, and Web of Science) up to 30 September 2018 and included all possible combinations of the following search terms: (diabetes mellitus OR Diabetes mellitus, type 2 OR diabetes mellitus, type 1) AND (epicardial fat tissue OR epicardial adipose tissue OR subepicardial adipose tissue OR subepicardial fat tissue). This study was limited to human studies published in English studies.

### Inclusion criteria

Identified studies were enrolled based on the following inclusion criteria: (1) DM patients were an experimental group and non-DM subjects were a control group; (2) quantitative measurement of EFT volume or thickness by echocardiography, cardiac magnetic resonance imaging or CT; (3) study of differences in EFT between DM patients and non–DM subjects; (4) the study reported a mean, standard deviation (SD), and sample size for DM patients and non-DM subjects; and (5) observational studies.

### Exclusion criteria

During the literature screening process, studies containing the following items were excluded: (1) experimental animal studies, reviews, or non-English literature; (2) the study did not provide sufficient information about the dataset (mean, SD and sample sizes).

### Data extraction and quality assessment

Two reviewers (Yingrui Li and Bin Liu) independently extracted data and assessed the quality of each study. If there was disagreement on a specific study, the two reviewers negotiated to reach a consensus. The data extracted from each study included the sample size and EFT of the experimental and control groups. Then, the Newcastle Ottawa scale (NOS) was used to assess the quality of the study. The scoring system consisted of three parts (population selection, comparability between groups, and exposure factors), and the results ranged from 0 to 9, with higher scores representing better methodology quality (Table 1).

**Table 1:** Characteristics of included studies

Study	Country	Arms	Diabetes type	N	Age (year)	BMI (Kg/m^2^)	EFT	Measurement tool	NOS Score
Chen et al. [27]	China	Diabetes	II	167	43.7 ± 10.9	22.6 ± 2.1	4.0 ± 1.5 mm	Echocardiography (end-diastole parasternal long axis)	7
		Non-diabetes		82	42.4 ± 10.2	22.0 ± 2.1	2.0 ± 1.5 mm		
Yazici et al. [29]	Turkey	Diabetes	I	36	30.8 ± 7.7	24.7 ± 3.8	3.3 ± 1.1 mm	Echocardiography (end-diastole parasternal average axis)	7
		Non-diabetes		43	29.9 ± 4.9	24.6 ± 3.0	2.3 ± 0.3 mm		
Iacobellis et al. [20]	American	Diabetes	I	15	52.8 ± 12.0	27.8 ± 5.2	7.2 ± 2.1 mm	Echocardiography (end-systole parasternal average axis)	7
		Non-diabetes		15	53.0 ± 9.0	27.4 ± 4.1	4.9 ± 2.5 mm		
Cetin et al. [21]	Turkey	Diabetes	II	139	54.3 ± 9.2	27.6 ± 3.1	6.0 ± 1.5 mm	Echocardiography (end-diastole parasternal average axis)	7
		Non-diabetes		40	52.1 ± 7.3	29.1 ± 4.2	4.4 ± 1.0 mm		
Seker et al. [33]	Turkey	Diabetes	II	186	62.5 ± 9.6	28.6 ± 4.4	6.5 ± 0.7 mm	Echocardiography (End-systole parasternal average axis)	7
		Non-diabetes		268	61.2 ± 10.9	27.6 ± 4.2	5.3 ± 1.0 mm		
Aslan et al. [37]	Turkey	Diabetes	I	76	30.6 ± 10.3	23.3 ± 2.7	3.6 ± 0.5 mm	Echocardiography (end-diastole parasternal average axis)	7
		Non-diabetes		36	32.4 ± 8.5	24.2 ± 2.7	3.0 ± 0.5 mm		
Wang et al. [32]	China	Diabetes	II	68	59.5 ± 9.9	26.9 ± 5.9	5.0 ± 1.2 mm	Echocardiography (end-diastole parasternal long axis)	7
		Non-diabetes		32	58.1 ± 9.2	23.7 ± 5.5	3.1 ± 0.8 mm		
Vasques et al. [30]	Brazil	Diabetes	II	31	45.0 ± 6.0	36.0 ± 4.7	1.0 ± 0.3 mm	Echocardiography (end-systole parasternal average axis)	7
		Non-diabetes		37	36.0 ± 11.0	27.9 ± 7.4	0.7 ± 0.2 mm		
Wang et al. [34]	China	Diabetes	II	49	56.8 ± 9.5	27.2 ± 3.6	166.1 ± 60.6 cm^3^	CT	8
		Non-diabetes		78	54.1 ± 6.5	25.4 ± 3.4	123.4 ± 41.8 cm^3^		
Keles et al. [35]	Turkey	Diabetes	I	45	34 ± 7.7	24.1 ± 3.4	7.0 ± 2.3 mm	Echocardiography (end-diastole parasternal long axis)	6
		Non-diabetes		35	32 ± 7.3	26.7 ± 6.5	6.0 ± 1.5 mm		
Akbas et al. [36]	Turkey	Diabetes	II	156	53.62 ± 9.33	31.21 ± 5.87	4.7 ± 1.6 mm	Echocardiography (end-diastole parasternal average axis)	7
		Non-diabetes		50	51.06 ± 8.74	32.86 ± 7.52	3.9 ± 1.6 mm		
Akyürek et al. [31]	Turkey	Diabetes	II	90	57.7 ± 11.4	28.9 ± 4.4	172.8 ± 64.9 cm^3^	CT	7
		Non-diabetes		62	55.6 ± 10.7	26.5 ± 2.9	68.9 ± 37.7 cm^3^		
Philouze et al. [28]	American	Diabetes	II	44	56.0 ± 6.0	26.9 ± 3.2	6.4 ± 1.7 mm	Echocardiography (end-systole parasternal average axis)	6
		Non-diabetes		35	52.0 ± 7.0	24.2 ± 3.6	3.3 ± 1.1 mm		

*BMI* body mass index, *EFT* epicardial fat tissue, *CT* computed tomography, *LDL* low-density lipoprotein, *HDL* high-density lipoprotein

### Data analysis

All statistical analyses were performed using Stata 12.0 using a fixed effect model or a random effects model to calculate the pooled standard mean difference (SMD) or weighted mean difference (WMD) for a 95% CI. All reported *P* values are two-sided with a significance level set at *P* < 0.05. The heterogeneity of each study was assessed by calculating the *χ*^2^ and I^2^ statistics. If *P* < 0.1 and I^2^ > 30%, a random effects model was applied; otherwise, a fixed effects model was applied [24]. Considering the potential impact of DM confounding factors on the results of the study, we performed a subgroup analysis of DM typing and EFT measurements. At the same time, we applied a sensitivity analysis to examine the impact of individual studies on the total merged effects to assess the reliability of the conclusions. We analyzed the symmetry of a funnel plot to evaluate possible small sample effects, and we used Begg’s test as well as Egger’s test to evaluate publication bias in the included studies. Statistical significance was considered at P < 0.10 [25]. If publication bias was detected, the Duval and Tweedie’s trim and fill method was applied using the random effects model to evaluate the adjusted effect scale [26]. Taking into account the differences between the studies, all our analyses used a random effects model.

Meta-regression is often used to explore the source and size of heterogeneity in a study and to further explain the impact of heterogeneity in a meta-analysis. We hypothesized that the included studies may have shown differences in cardiovascular disease risk factors (BMI, dyslipidemia, waist hip rate) in DM patients. To assess the possible effects of these variables on the different outcomes observed in the meta-analysis, we established a regression model with the EFT value as the dependent variable (y) and the abovementioned covariate as the independent variable (x).

In a conventional meta-analysis, repeated significance testing of cumulative data increases the overall risk of type I error; however, TSA can reduce the risk of type I error and estimate the required information size (RIS) needed to achieve a preset power level, draw benefit boundaries and harm boundaries, and calculate futility. An RIS used with boundaries can infer whether further studies are needed [28]. We conducted the TSA with a 5% risk of a type I error and a power of 80% as well as an a-spending adjusted 95% CI for repetitive significance testing.

### Availability of data and materials

All the data were extracted from public sources.

## Results

### Description of the studies

**Fig. 1:**
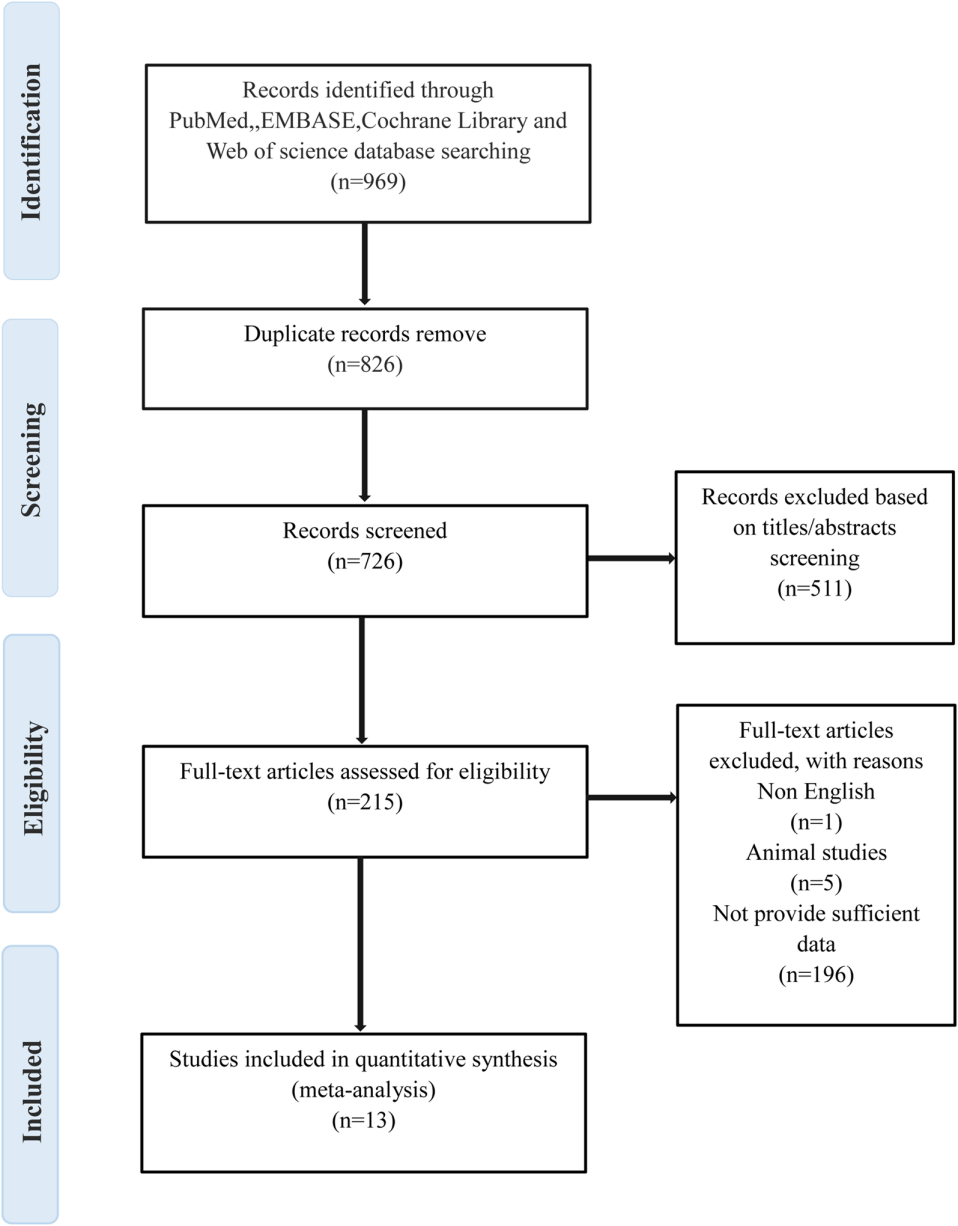
Flowchart of the study selection process used to identify studies to be included in the meta-analysis

Our search strategy yielded a total of 969 studies. After excluding repeated studies, there were 726 studies, of which 511 were decided to be irrelevant to our review topic after confirming the title or abstract. A further 196 studies lacked sufficient data, 5 were studies of animals, and 1 was excluded because it was not published in English. Therefore, our final analysis included 13 studies (1102 patients with DM and 813 healthy control subjects) that measured EFT in a DM group and a control group [20, 21, 27–37] (Fig. 1).

The main features of the studies are shown in Table 1. The number of patients in each study ranged from 15 to 186. EFT thickness was measured by echocardiography in 11 studies [20, 21, 27–30, 32, 33, 35–37], and the volume of EFT was measured by CT in 2 studies [31–34]. We assessed the characteristics and quality of these studies by NOS score.

### DM group versus control group

**Fig. 2:**
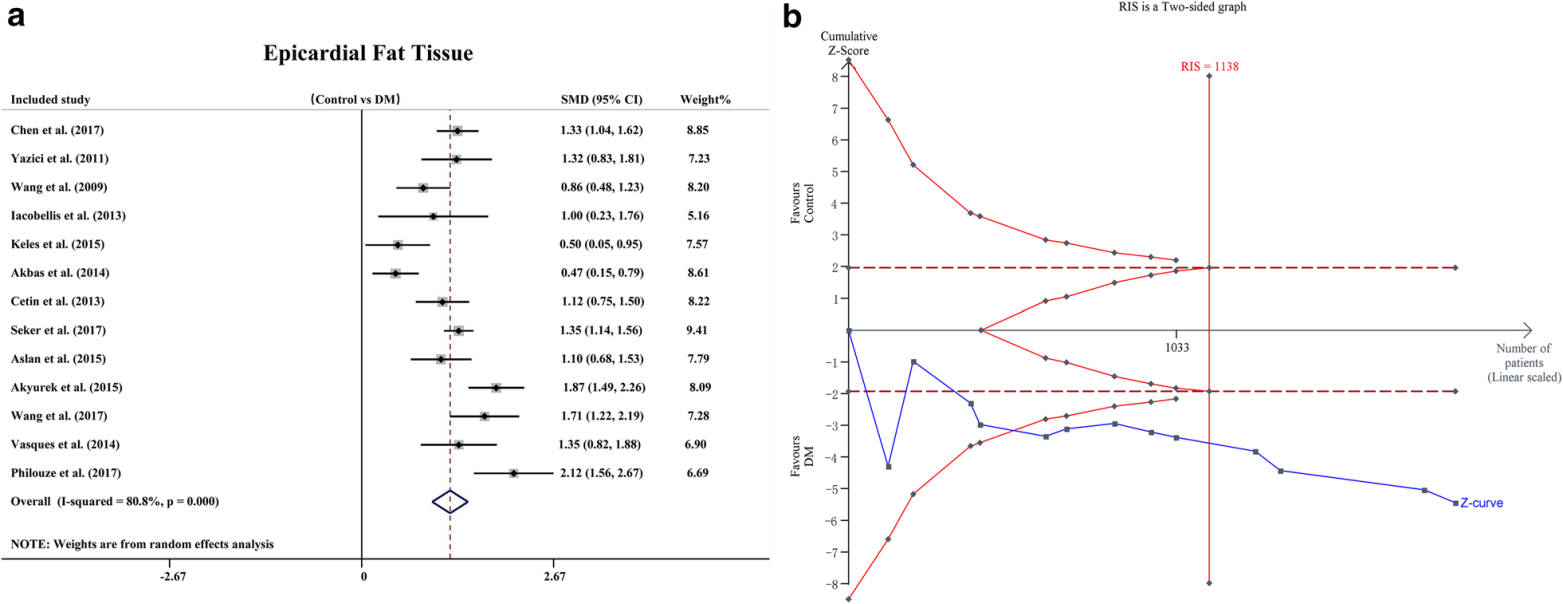
SMD in EFT between patients with DM and the non-DM control group. **a** Meta-analysis; **b** trial sequential analysis

EFT was reported in all studies and was significantly higher in 1102 patients with DM than in 813 control subjects (SMD: 1.23; 95% CI 0.98, 1.48; *P* = 0.000, Fig. 2a). There was significant heterogeneity among the studies (I^2^ = 80.8%, *P* = 0.000). The TSA showed that the cumulative z-curve crossed the boundary for futility (TSA-adjusted 95% CI 0.91, 2.13; *P *< 0.0001, Fig. 2b).

**Fig. 3:**
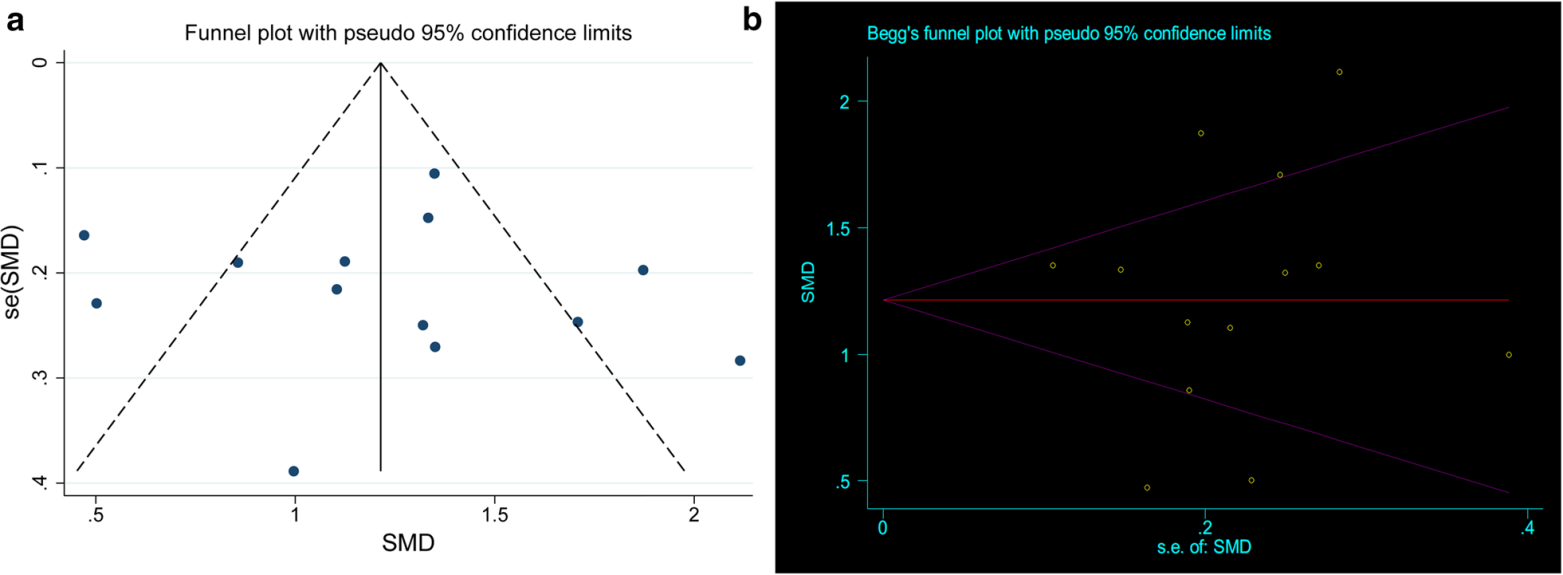
Evaluation of publication bias by funnel plot and Begg’s test. **a** Visual evaluation of the funnel plot on enrolled DM patients and the non-DM control group; **b** Begg’s test to assess publication bias in the included studies

Publication bias in the included studies may have affected the results of our meta-analysis. Therefore, we used a funnel plot, Begg’s test and Egger’s test to assess potential publication bias in the included studies. Less publication bias was observed in our evaluation of the funnel plots for the DM and control groups (Fig. 3a), and this finding was confirmed by Egger’s test (*P *= 0.869) and Begg’s test (*P *= 0.855, Fig. 3b).

We performed a sensitivity analysis by deleting individual studies one by one and performing an additional meta-analysis with each study removed. We evaluated the effect of each deletion on the pooled SMD. Based on the sensitivity analysis results, we observed that none of the studies affected the overall effect, indicating that our meta-analysis is statistically stable.

**Fig. 4:**
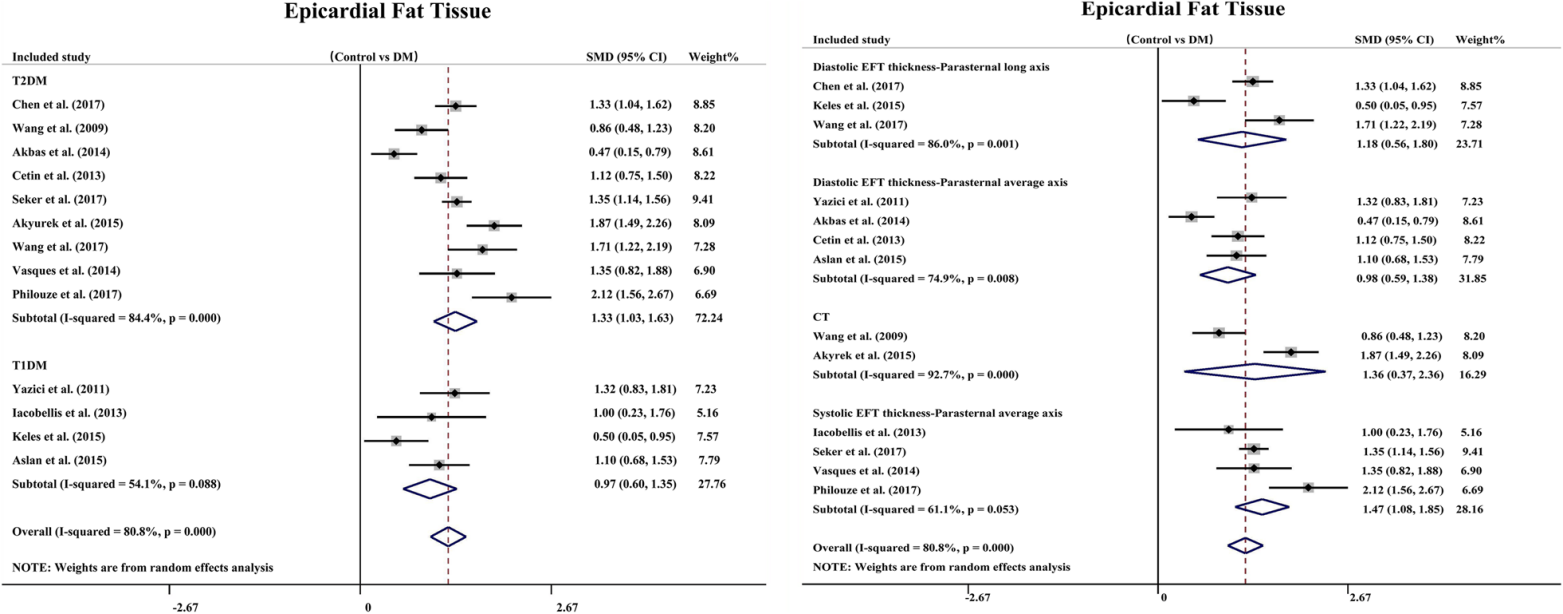
Subgroup analysis of SMD in EFT between patients with DM and the non-DM control group: **a** subgroup analysis by DM typing (T1DM or T2DM); **b** subgroup analyses by different measurements of EFT (CT or ultrasonic)

To eliminate the impact of confounding factors on the results of this study, we conducted two subgroup analyses. We divided the included studies into a T1DM group and a T2DM group according to the difference in DM typing and divided the included studies into a CT measurement group, an ultrasonic parasternal average axis measurement of end systolic EFT thickness group, an ultrasonic parasternal average axis measurement of end diastolic EFT thickness group and an ultrasonic parasternal long axis measurement of end diastolic EFT thickness group according to the difference in EFT measurement. The subgroup analysis indicated there was heterogeneity in the T1DM patients (n = 4) (SMD: 0.97; 95% CI 0.60, 1.35; *P *= 0.000) (I^2^ = 54.1%, P = 0.088, Fig. 4a), and ultrasonic parasternal average axis measurement of end systolic EFT thickness (n = 4) (SMD: 1.47 mm; 95% CI 1.08, 1.85; *P *= 0.000) (I^2^ = 61.1) %, P = 0.053, Fig. 4b) showed a significant decrease. Moreover, EFT was higher in the DM patients in each subgroup than in the non-DM patients, and the total effect was statistically significant.

**Fig. 5:**
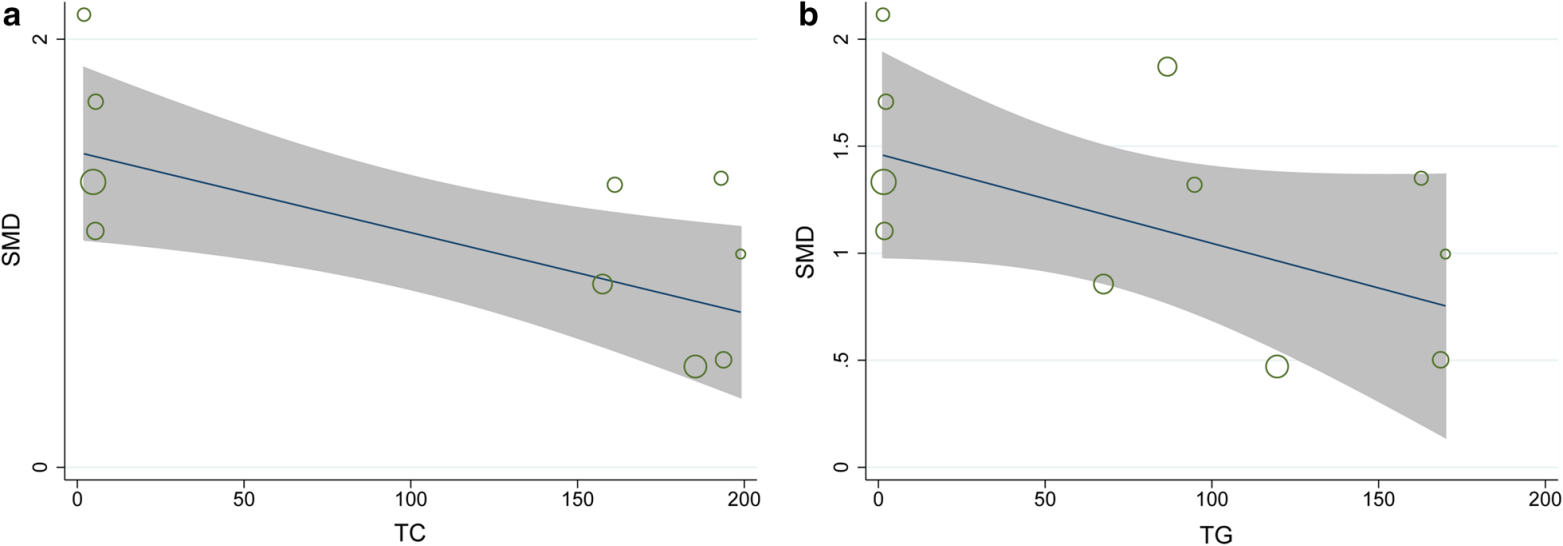
Meta-regression analyses of SMD in EFT between patients with DM and the non-DM control group: **a** effect of TC on EFT; **b** effect of TG on EFT

To evaluate other factors that may have affected the results, we conducted a regression analysis of the included studies. Our regression analysis showed that TC levels significantly affected EFT, suggesting that TC is the source of heterogeneity (*P* = 0.011, τ^2^ = 0.0665, Adj R-squared = 67.31%, I^2^ res = 55.97%, Fig. 5a). Based on regression models studied in DM patients and control subjects, we found that TG (*P* = 0.099, τ^2^ = 0.1771, Adj R-squared = 22.80%, I^2^ res = 79.58%, Fig. 5b) was a confounding factor that significantly affected EFT. Other risk factors, including BMI (P = 0.732, τ^2^ = 0.1999, Adj R-squared = − 10.57%, I-squared res = 81.70%), low Density Lipoprotein (LDL, P = 0.120, τ^2^ = 0.1886, Adj R-squared = 17.75%, I-squared res = 81.33%), high density lipoprotein (HDL, P = 0.145, τ^2^ = 0.1955, Adj R-squared = 14.75%, I-squared res = 81.40%) and systolic blood pressure (P = 0.963, τ^2^ = 0.2362, Adj R-squared = − 17.10%, I-squared res = 85.34%), did not affect the results.

## Discussion

This meta-analysis included 13 studies that contained 1102 patients with DM and 813 healthy subjects. Our analysis confirmed that DM is associated with an increase in EFT. Furthermore, our TSA results indicated that the available samples were sufficient and confirmed that firm evidence was reached; the sensitivity analysis further confirmed these findings. Regression analysis indicated that TC and TG levels significantly affected our results, and a subgroup analysis suggested that there were more abnormal EFTs in DM patients with different DM types and EFT measurements than in non-DM patients.

In recent years, research on the relationship between EFT and DM has gradually increased, but the reasons for the increase in EFT observed in DM patients has not been studied. Possible causes include decreased physical activity and an increased food supply, which lead to adipose tissue deposition in T2DM patients [38]. Additionally, genes associated with lipid metabolism have been shown to be missing, dislocated and mutated in DM patients [39]. Finally, T2DM patients regulate the distribution of sex steroids in body fat tissue (such as androgen) abnormally [40]. Previous studies have shown that EFT is closely related to insulin levels in serum in addition to Resistin mRNA levels and may be involved in the formation of insulin resistance. A significant increase in EFT has also been observed in chronic systemic inflammation, such as obesity and hyperlipidemia. It has been suggested that it is also involved in abnormal lipid metabolism in the body [41, 42].

To better understand the relationship between EFT and DM, a meta-analysis was conducted to explore the amounts of EFT in DM patients and non-DM patients. The results showed that the volume of EFT was significantly higher in DM patients with significant heterogeneity. To explore the source of heterogeneity, we conducted a subgroup analyses of different DM typing and EFT measurement methods. The heterogeneity in T1DM and the subgroup measuring EFT thickness during the systolic period decreased significantly, suggesting that different DM typing and different EFT measurement methods may be potential sources of heterogeneity.

T1DM and T2DM have very different pathogenesis and pathological processes, and EFT plays a specific role in both types of DM. Under pathological conditions, EFT can secrete a variety of bioactive adipokines and pro-inflammatory factors, such as TNF-alpha, IL-6, resistin, visfatin, omentin, and leptin [43–47]. Previous studies have suggested that abnormal increases in visceral fat may play a major role in the development of insulin resistance and may therefore further promote DM [48, 49]. In a study by Galletti et al. leptin inhibited insulin synthesis and secretion and increased insulin sensitivity [50]. It has also been suggested that changes in leptin secretion or sensitivity may be related to the pathogenesis of T1DM, although these findings remain controversial [50, 51]. The results of our subgroup analysis suggest that EFT is increased in both T1DM and T2DM, indicating that in the diagnosis and treatment of DM, EFT could be used as a new research target to promote the study of DM and provide new ideas for the clinical treatment of T1DM and T2DM.

EFT can be detected by ultrasound, CT and CMR, and EFT thickness measured by ultrasound has recently become a simple practical tool for cardiovascular risk stratification [9]. Previous studies have suggested that magnetic resonance imaging is the gold standard for estimating systemic adipose tissue [52, 53]. CMR and CT are not limited by the position and direction of the imaging plane, and it is therefore more accurate to evaluate the absolute amount of EFT compared with the ultrasound, which uses a single point measurement of EFT to assess the absolute amount of EFT. However, in the study by Iacobellis et al. EFT assessment of the right ventricular free wall by echocardiography showed an excellent correlation with the EFT measurements obtained by CMR [8]. The results of our subgroup analysis showed that the volume of EFT was higher in DM patients than in non-DM patients regardless of the method of EFT measurement, suggesting that there was no significant difference among ultrasound, CT and CMR in the measurement of abnormal EFT increases in DM patients in clinical practice.

Epicardial adipose tissue is an extremely active adipose tissue with unique biological molecular and anatomical characteristics. It is not only a passive lipid storage unit but also an active participant in lipid and energy homeostasis in the myocardium. One of the biggest differences between EFT and other visceral fat is its greater ability to release and synthesize free fatty acids (FFA) [12, 54]. Under physiological conditions, EFT exerts a cardioprotective effect through its anti-atherosclerosis/anti-inflammatory properties as well as its high FFA release and uptake rates [55]. However, abnormally increased EFT can secrete a variety of bioactive substances and excessive fatty acids, which can lead to systemic inflammation, insulin resistance, abnormal blood lipid indexes, such as TC and TG, and ultimately lead to DM, metabolic syndrome and atherosclerosis [56, 57]. Therefore, a meta-regression analysis was performed to evaluate other factors (BMI, LDL, HDL, TC, TG, and systolic blood pressure) that might have had an impact on the results. The results suggested that TC and TG affected the results of this study, while other risk factors did not.

Our study indicates that compared with non-DM patients, in DM patients, the amount of EFT is significantly increased, and this has considerable clinical significance for providing new ideas for the diagnosis and treatment of DM. In a study by Kang et al. 321 CAD patients who received high-dose statins were retrospectively studied, and the results showed that EFT thickness during the systolic period was an independent predictor of new DM in patients with coronary heart disease treated with high-intensity statins [58]. At the same time, Chun et al. found that there was a significant correlation between left ventricular EFT thickness and the prevalence of diabetes in Korean men [59]. In the studies by Bouchi et al. and Sato et al., the SGLT-2 inhibitors Dapagliflozin and Luseogliflozin both improved systemic inflammation and were used for EFT, thereby reducing the EFT volume of DM patients and thus reducing the risk of cardiovascular disease [60, 61]. However, the clinical efficacy of these new therapies has not yet been confirmed, and further studies are needed [62]. Previous research results are consistent with our meta-analysis results, but previous studies did not consider the impact of other confounding factors on the results. Our results suggest that even among different types of DM or different EFT measurement techniques, patients with DM always have an abnormal increase in EFT compared with patients without DM, suggesting that the abnormal increase of EFT can be used as an independent predictor of new DM and a new target for DM drug therapy.

## Limitations

We should consider some potential limitations of this study. First, five studies included [20, 28–30, 35] were small sample size. Second, there are limited studies in some subgroup analysis, and more studies are needed to support these results. Finally, it is important to assess heterogeneity among studies, and although it may not be possible to identify all possible sources of heterogeneity, the stability of our outcomes was confirmed after adjusting for potential publication bias.

## Future directions

Our study indicates that DM patients have more EFT than non-DM patients, and this provides new ideas for the diagnosis and treatment of DM in the future and research into DM performed in the future. EFT is closely related to the occurrence of CHD [63, 64]. The difference in EFT between DM patients and non-DM patients may have an important relationship with the incidence of cardiovascular diseases, but whether EFT can be used to assess the risk of cardiovascular diseases in DM patients at an early stage needs to be confirmed by further studies.

## Conclusions

In general, our meta-analysis suggests that the amount of EFT is higher in DM patients than in non-DM patients regardless of T1DM or T2DM status or the EFT measurement method used. This suggests that an abnormal increase in EFT can be used as an independent predictor of new DM and a new target for DM drug therapy.

## Abbreviations

EFT: epicardial adipose tissue
DM: diabetes mellitus
T1DM: type 1 diabetes mellitus
T2DM: type 2 diabetes mellitus
TSA: trial sequential analysis
TC: total cholesterol
TG: triglycerides
GDM: gestational diabetes mellitus
CT: computed tomography
SD: standard deviation
NOS: Newcastle Ottawa Scale
SMD: standard mean difference
WMD: weighted mean difference
RIS: required information size
